# Prevalence and clustering of metabolic risk factors for type 2 diabetes among Chinese adults in Shanghai, China

**DOI:** 10.1186/1471-2458-10-683

**Published:** 2010-11-09

**Authors:** He Xu, Yiqing Song, Nai-Chieh You, Zuo-Feng Zhang, Sander Greenland, Earl S Ford, Lin He, Simin Liu

**Affiliations:** 1 Institute for Nutritional Sciences, Shanghai Institute for Biological Sciences, Chinese Academy of Sciences, Shanghai, China; 2 Shanghai Bio-X center, Jiaotong University, Shanghai, China; 3 Program on Genomics and Nutrition, Department of Epidemiology, University of California, Los Angels (UCLA), Los Angeles, CA, USA; 4 Division of Preventive Medicine, Brigham & Women's Hospital, Harvard Medical School, Boston, MA, USA; 5 Center for Metabolic Disease Prevention and Department of Medicine, UCLA, Los Angeles, CA, USA; 6 Departments of Epidemiology and Statistics, UCLA, Los Angeles, CA, USA; 7 Division of Adult and Community Health, Centers for Disease Control and Prevention (CDC), Atlanta, GA, USA

## Abstract

**Background:**

Type 2 diabetes is becoming an epidemic in China. To evaluate the prevalence, clustering of metabolic risk factors and their impact on type 2 diabetes, we conducted a population-based study in Shanghai, China's largest metropolitan area.

**Methods:**

From 2006 to 2007, 2,113 type 2 diabetes cases and 2,458 comparable controls of adults aged 40 to 79 years were enrolled. Demographic, lifestyle, and dietary factors were assessed via standardized questionnaires. Plasma, red and white blood cells were collected and stored for future studies. Anthropometric indices and biochemical intermediates (including blood pressure, fasting glucose, glycosylated hemoglobin, and blood lipids) were measured. The prevalence of metabolic syndrome were also compared following two criteria recommended by the Chinese Diabetes Society (CDS, 2004) and the National Cholesterol Education Program's Adult Treatment Panel III (ATP III, 2002).

**Results:**

Prevalence of metabolic syndrome (62% vs. 15% using CDS criteria) and its individual components, including obesity (51% vs. 42%), hypertension (54% vs. 41%), hypertriglyceridemia (42% vs. 32%), and low high-density lipoprotein-cholesterol (HDL) levels (36% vs. 25%) were higher in diabetes cases than controls. Regardless of criteria used, those with impaired fasting glucose (IFG) had similarly high prevalence of metabolic syndrome as did diabetes cases. In a multiple logistic regression model adjusted for demographics and lifestyle risk factors, the odds ratios of diabetes (95% CI) were 1.23 (1.04-1.45) for overweight (28 >= BMI >= 24), 1.81 (1.45-2.25) for obesity (BMI > 28), 1.53 (1.30-1.80) for central obesity (waist circumference > 80 cm for woman or waist circumference > 85 cm for man), 1.36 (1.17-1.59) for hypertension (sbp/dbp >= 140/90 mmHg), 1.55 (1.32-1.82) for high triglycerides (triglycerides > 1.70 mmol/l) and 1.52 (1.23-1.79) for low HDL-C (HDL-C < 1.04 mmol/L).

**Conclusions:**

These data indicate that multiple metabolic risk factors--individually or jointly--were more prevalent in diabetes patients than in controls. Further research will examine hypotheses concerning the high prevalence of IFG, family history, and central obesity, aiding development of multifaceted preventive strategies specific to this population.

## Background

In recent decades, rapid industrialization and urbanization in the People's Republic of China has led to major changes in patterns of complex diseases (e.g. type 2 diabetes), which are rising to epidemic proportions. According to the World Health Organization (WHO), the number of patients worldwide with diabetes will reach 366 million by 2030, with 42 million of them residing in China [1], especially in metropolitan regions. In Shanghai, China's largest city, the prevalence of type 2 diabetes in 2006 was ~9%, far exceeding the average of ~3% nationwide [2]. In 2010, Yang and colleagues [3] reported that the prevalence of diabetes in China has now reached 9.7% among adults (> 20 years old).

Although metabolic risk factors such as obesity, glucose intolerance, hypertension, and dyslipdemia (high triglyceride and low high-density lipoprotein-cholesterol) have become more prevalent, much remains to be learned about the impact of these risk factors on type 2 diabetes risk in populations that have recently undergone marked socioeconomic and nutrition transition. Compared to Caucasians in Western cultures, for example, Chinese people consume much more cereal-based products, which appear to confer greater risk of type 2 diabetes due to high glycemic index and/or glycemic load [4]. Moreover, while the clustering of metabolic risk factors is well recognized among high risk individuals, few studies have examined whether different definitions of metabolic syndrome yield similar estimates for individuals at high risk for diabetes, such as those with IFG, who are likely to benefit from early intervention strategies.

More recently, genome-wide association studies have identified some genetic variants for increased type 2 diabetes susceptibility among Caucasians [5]. These genetic variants appeared to exhibit modest effects and may exert their full impact on diabetes risk in the presence of certain environmental exposures, making inferences of the genetic associations to other racial and ethnic populations difficult. Thus, it is important to identify both environmental and genetic factors that affect diabetes risk directly in Chinese. To answer many questions in diabetes research that are of fundamental importance toward the ultimate control of this epidemic, we conducted a large population-based case-control study in Shanghai, China. In particular, we aimed to 1) examine the prevalence and distribution of risk factors for type 2 diabetes in Chinese adults living in Shanghai, 2) investigate the risk of metabolic syndrome among those with impaired fasting glucose and in apparently healthy non-diabetic individuals with emphasis on comparing MetS prevalence between the CDS and NCEP ATP III criteria, and 3) generate an epidemiologic resource to investigate hypotheses relating gene-environment interactions to type 2 diabetes risk and its complications in follow-up effort.

## Methods

In 2000, the Centers for Disease Control and Prevention of Pudong and Baoshan districts developed a health registration system for local residents and subsequently implemented a management system for type 2 diabetic patients who had been diagnosed according to the World Health Organization (WHO) criteria [6]. Between December 2006 and August 2007, the total number of registered type 2 diabetic patients was approximately ~3,000 in Pudong and ~3,500 in Baoshan. We randomly chose 6 communities (by different economic status, three communities each in Pudong and Baoshan) and contacted 2,401 cases who met our inclusion criteria (88% responded). Controls were identified from the population registers using the same inclusion and exclusion criteria for cases in the same communities from which cases occurred. A total of 3,234 non-diabetic individuals were randomly selected and invited by letter or telephone to participate in the study. 76% of these responded. All eligible type 2 diabetic patients and comparable controls were identified to participate in this Shanghai Diabetes Study (SDS) where dietary, anthropometrical, and biochemical assessments were conducted. Written informed consents were obtained from all participants. This study was approved by both local authorities and the Ethics Committee of Shanghai Institute for Biological Sciences (ER-SIBS-250701), and the UCLA Institutional Review Board (UCLA IRB #06-05-096-11).

### Participant enrollment and data collection

Of approximately 6,500 type 2 diabetes cases available in Baoshan and Pudong, 2,113 type 2 diabetes patients were enrolled in the SDS. They met the following inclusion criteria in the SDS: 40 - 79 years old; of Chinese Han ethnicity; had resident registration records, and lived locally >= 5 years. We excluded participants who had physical disability, severe diseases (e.g. cancer and stroke) during the previous 6 months, or who were unable or unwilling to sign the informed consent form. Aside from the fact that both districts have a diabetes surveillance system, the two independent sets of cases in a well-established health-care network were intended to serve as split-replication samples to each other (to confirm any new findings that may emerge from future genetic studies). The same inclusion and exclusion criteria for cases were also applied to the selection of controls in these two districts, ultimately enrolling 2,458 controls. These apparently healthy were then examined to determine if they had ever been diagnosed with diabetes or if their fasting glucose levels met the WHO criteria for type 2 diabetes diagnosis. 308 individuals were identified to have impaired fasting glucose (IFG, fasting plasma glucose levels from 6.1 mmol/L to 6.9 mmol/L).

All enrollees were examined by centrally-trained staff at clinics for collection of participant information, including a questionnaire, anthropometric indices and overnight fasting (>= 8 hours) blood samples.

### Lifestyle, dietary, and anthropometric measurements

All participants completed a standardized questionnaire that included questions about their demographic characteristics, history of chronic diseases, family history of diabetes in first-degree relatives, cigarette smoking, alcohol consumption, and physical activity. Cigarette smoking was defined as "at least once daily," alcohol consumption was defined as "regularly drink more than 50 ml each time and at least twice/week". Low, medium and high levels of physical activity were defined as "daily housework, walking, flower planting and light stretch activity," "jogging, swimming and ping-pong" and "hiking, tennis and exercise in gym" respectively. For diabetes patients, diabetic symptoms, onset time, and hypoglycemic medications were also collected.

A food frequency questionnaire (FFQ) was also administered to rank participants according to the distribution of long-term nutrient and food intake in the SDS. The development and validation of a similar FFQ used for Han Chinese in Shanghai have been reported previously[7,8]. In general, the FFQ can reasonably categorize usual intake of nutrients and food groups among Chinese adults. Briefly, our FFQ includes 100 food-items in 10 categories (staple foods, meats, aquatic products, beans and related products, eggs, milks, vegetables, fruits, pickled vegetables and others). For each food item, participants were asked how frequently in reference to the previous year ("never", "times per day", "times per week", "times per month", and "times per year") and the quantities they consumed the specific item.

All measurements were conducted using a standardized protocol. After an overnight fast, participants were asked to sit at ease, rest for ≥ 5 minutes, and avoid smoking and drinking alcoholic beverages and coffee prior to the scheduled appointment. Blood pressure measurements 30 seconds apart were taken from the participant's right arm, using a conventional mercury sphygmomanometer with appropriate cuff size. The average value of two consecutive measurements was recorded. Subjects taking antihypertensive medications and those with systolic blood pressure ≥ 140 mm Hg, diastolic blood pressure ≥ 90 mm Hg (WHO/ISH) were defined as hypertensive. Height (in centimeter, cm), weight (in kilogram, kg), waist circumference, and hip circumference were measured after participants took off their shoes, hats, coats, and sweaters. Waist circumference was measured at the midpoint between the inferior costal margin and the superior border of the iliac crest on the midaxillary line and hip circumference was measured at the maximum extension of the buttocks. Body mass index (BMI) was defined as kg/m^2^. Following the recommendation by the Working Group on Obesity in China (International Life Science Association, 2001), we defined central obesity using either waist circumference for men >= 85 cm, for women >= 80 cm or waist-hip ratio <= 0.75, 0.76~0.85, >= 0.86. BMI was defined as < 24 for normal, 24~28 for overweight and > 28 for obesity.

### Laboratory and biochemical measurements

Fasting blood specimens were collected using vacuum negative pressure tubes. Blood glucose, glycosylated hemoglobulin A1C (HbA1c), and lipids were measured using Roche modular P800 autoanalyzer. All measurements were performed at the biochemistry and immunology laboratory of Dongfang Hospital, a teaching affiliate of Tongji University. The interassay coefficients of variation were 1.7% for glucose, 3.2% for HbA1c, 1.8% for triglycerides, 1.7% for total cholesterol, 1.2% for LDL-cholesterol, and 1.3% for HDL-cholesterol. The definition of abnormal lipid profiles was triglycerides <= 1.70 mmol/L or HDL-Cholesterol >= 1.04 mmol/L (Chinese Cardiovascular Disease Association, 1997).

### Definition of metabolic syndrome

We defined metabolic syndromes using two different criteria (Chinese Diabetes Society, CDS, 2004) [9] and the National Cholesterol Education Program's Adult Treatment Panel III (NCEP ATP III, 2002) [10]. According to the CDS criteria, a participant has metabolic syndrome if he or she has three or more of the following criteria: BMI >= 25 kg/m2; fasting glucose >= 110 mg/dl or 2-h plasma glucose >= 140 mg/dl or diabetes; blood pressure >= 140/90 mmHg or hypertensive; and triglycerides >= 150 mg/dl or HDL cholesterol < 35 mg/dl in men or < 39 mg/dl in women. The metabolic syndrome was also defined by the NCEP ATPIII, when three or more of the following five risk determinants were present: waist circumference (men > 102 cm, women > 88 cm), triglycerides >= 150 mg/dl, HDL-C (men < 40, women < 50 mg/dl), blood pressure (>= 130/>= 85 mmHg), and fasting glucose >= 110 mg/dl.

### Statistical analysis

We first examined differences in age, sex, education, age at diagnosis, duration of diabetes, family history and hypoglycemia medication by two recruiting districts. The crude medians (ranges) and prevalence of demographic, lifestyle, anthropometric, and biochemical characteristics by outcome status (type 2 diabetes, IFG, and controls) were then calculated and compared using either Kruskal-Wallis test (for continuous) and χ^2 ^test (for categorical variables). We also estimated the prevalence of metabolic syndrome according to different criteria and its individual components by outcome status. Odds ratios (ORs) and 95% confidence intervals (CIs) of type 2 diabetes by demographics and lifestyle risk factors were calculated in a multiple logistic regression model. Finally, we calculated ORs and 95% CIs of type 2 diabetes by components of metabolic syndrome in a multiple logistic regression model adjusted for age, gender, education, family history, smoking status, alcohol drinking and leisure physical activities. All p values were two-sided, and all statistical analyses were conducted using SAS (version 9.2; SAS institute, Cary, NC).

## Results

After data cleaning, 2,050 cases and 2,418 controls' information were analyzed. Of the 4,468 participants in the SDS, 1,763 (913 cases and 850 controls) were from Pudong and 2,705 (1,137 cases and 1,568 controls) from Baoshan. There were no apparent differences in basic demographic characteristics between the two districts (data in Additional File 1), except that participants from Baoshan reported higher levels of education than did those from Pudong.

**Figure 1:**
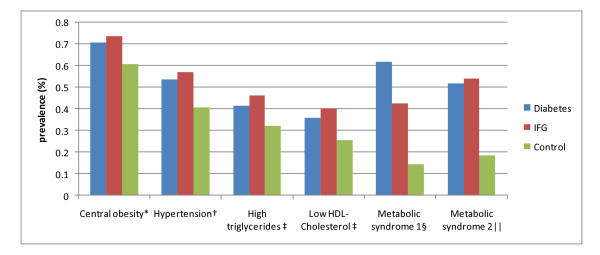
**Prevalence of metabolic syndrome and its individual components by outcome status among 4,468 participants in the Shanghai Diabetes Study**. * Criteria based on waist circumference according to the Working Group on Obesity in China, International Life Science Association. 2001: men >= 85 cm and women >= 80 cm. †Criteria according to the World Health Organization/International Society of Hypertension (WHO/ISH). 1999. ‡Criteria according to Chinese Cardiovascular Disease Association. 1997: Triglycerides <= 1.70 mmol/L; HDL-Cholesterol >= 1.04 mmol/L. § Criteria according to Chinese Diabetes Society, 2004 (three or more of the following conditions): 1. BMI ≥ 25.0 kg/m2; 2. fasting glucose ≥ 110 mg/dl (6.1 mmol/L) or 2-h plasma glucose ≥ 140 mg/dl (7.8 mmol/L) or diabetes patients; 3. Blood pressure: ≥ 140/90 mmHg or Hypertension patients; 4. triglycerides ≥ 150 mg/dl or HDL cholesterol: men < 35 mg/dl, women < 39 mg/dl. || Criteria according to National Cholesterol Education Program's Adult Treatment Panel III (ATP III, 2002) (three or more of the following conditions): 1. abdominal obesity: waist circumference: men > 102 cm, women > 88cm); 2. triglycerides >= 150 mg/dL; 3. HDL cholesterol: men < 40 mg/dL, women < 50 mg/dL; 4. blood pressure  135/85 mm Hg; 5. fasting glucose >= 110 mg/dL.

Type 2 diabetes cases had a higher prevalence of traditional risk factors than controls (Table 1: 31% vs. 11% for family history of diabetes, 31% vs. 19% for less than 6 years education, 25.1 vs. 24.3 for BMI, 87 vs. 83 for waist circumference, 0.89 vs. 0.86 for waist-hip ratio, 139 mmHg vs. 130 mmHg for systolic blood pressure, 7.5 mmol/L vs. 4.9 mmol/L for glucose, 7.1% vs. 5.8% for HBA1C, 1.53 mmol/L vs. 1.35 mmol/L for triglycerides, 1.13 mmol/L vs. 1.22 mmol/L for HDL-cholesterol). The overall prevalence of metabolic syndrome were 62% using CDS and 52% using NCEP ATP III in type 2 diabetes cases, 43% and 54% in participants with IFG, and 15% and 19% in controls, respectively (Figure 1). Each individual component of metabolic syndrome was also more prevalent in diabetes cases than controls (Figure 1). The occurrence of central obesity, hypertension, hypertriglyceridemia, and low HDL-cholesterol was similar between diabetes cases and IFG individuals.

**Table 1:** Selected demographic, lifestyle, anthropometric, and biochemical characteristics by outcome status among 4,468 participants in the Shanghai Diabetes Study

Characteristics	Diabetes (n = 2,050)	IFG (n = 308)	Control (n = 2,110)	**P value**†
Age (years) *	63 (40-79)	63 (41-79)	59 (40-79)	< 0.0001
40-49, %	5	4	12	< 0.0001
50-59, %	30	32	45	
60-69, %	38	39	30	
70-79, %	27	25	12	
Sex (female, %)	59	65	70	< 0.0001
Education (years, %)				< 0.0001
0-6	31	30	19	
7-9	54	50	62	
≥ 10	15	20	19	
Family history of diabetes, %	31	12	11	< 0.0001
				
Smoking status, %				< 0.0001
Current	17	12	14	
Never	77	84	83	
Past	6	4	4	
				
Alcohol drinking, %				< 0.0001
Current	13	13	14	
Never	83	86	85	
Ex-drinker	4	2	1	
				
Leisure physical activity, %				0.02
Low	63	59	67	
Moderate	36	41	33	
High	1	0	1	
BMI (kg/m2)*	25.1 (13.1-43.4)	25.1 (16.5-37.2)	24.3 (15.4-42.1)	< 0.0001
Waist (cm)*	87 (59-125)	86 (62-119)	83 (56-115)	< 0.0001
Waist-hip ratio*	0.89 (0.62-1.25)	0.89 (0.68-1.11)	0.86 (0.64-1.10)	< 0.0001
SBP (mmHg)*	139 (90-218)	140 (90-192)	130 (82-213)	< 0.0001
DBP (mmHg)*	80 (54-130)	82 (56-110)	80 (48-120)	0.001
Glucose (mmol/l)*	7.5 (2.5-23.1)	6.1 (3.6-7.0)	4.9 (2.1-7.8)	< 0.0001
HBA1c (%)*	7.1 (0.0-17.5)	6.2 (4.3-8.0)	5.8 (0.0-12.6)	< 0.0001
Total Cholesterol (mmol/l)*	4.44 (0.93-11.91)	4.43 (2.81-8.53)	4.47 (2.03-11.32)	0.31
Triglycerides (mmol/l)*	1.53 (0.50-22.75)	1.62 (0.46-12.65)	1.35 (0.09-21.10)	< 0.0001
LDL-Cholesterol (mmol/l)*	2.72 (0.39-6.81)	2.80 (1.31-5.64)	2.75 (0.54-8.64)	0.004
HDL-Cholesterol (mmol/l)*	1.13 (0.00-2.60)	1.10 (0.58-2.05)	1.22 (0.00-2.74)	< 0.0001

*Data are presented as the median and range (in parentheses).

† P value for difference by outcome status.

As expected, education, family history of diabetes and all components of metabolic syndrome, were each independently associated with risk of type 2 diabetes. In a multiple logistic regression model where all demographics and lifestyle variables were simultaneously adjusted, the ORs (95% CI) were 0.68 (0.48-0.96) for education (> 10 vs. 0-6 years), 5.24 (4.21-6.52)for family history of diabetes (yes vs. no) (Table 2), 1.23 (1.04-1.45) for overweight (28 >= BMI >= 24 vs. BMI < 24), 1.81 (1.45-2.25) for obesity (BMI > 28 vs. BMI < 24), 1.53 (1.30-1.80) for central obesity (waist circumference > 80 cm for woman or waist circumference > 85 cm for man), 1.36 (1.17-1.59) for hypertension (SBP/DBP >= 140/90 mmHg), 1.55 (1.32-1.82) for high triglycerides (triglycerides > 1.70 mmol/l) and 1.52 (1.23-1.79) for low HDL-C (HDL-C < 1.04 mmol/L) (Table 3). These independent associations between individual risk factor and diabetes risk did not differ by overall obesity status (data in Additional File 2).

**Table 2:** Odds ratios (ORs) and 95% confidence intervals (CIs) of type 2 diabetes by demographics and lifestyle risk factors among 4,160 participants in the Shanghai Diabetes Study*

Factors	No. of cases/controls	OR (crude 95%CI)	**OR (adjusted 95%)**†
Age (years)			
40-49	111/257	1.00	1.00
50-59	605/958	1.46 (1.14-1.87)	1.46 (1.07-1.99)
60-69	779/633	2.85 (2.23-3.64)	2.86 (2.09-3.92)
>= 70	555/262	4.90 (3.75-6.40)	4.81 (3.41-6.77)
Sex			
female	1,211/1,467	1.00	1.00
male	839/643	1.58 (1.39-1.80)	1.45 (1.17-1.80)
Education (years)			
0-6	639/392	1.00	1.00
7-9	1,270/1,569	0.50 (0.43-0.58)	0.72 (0.59-0.86)
>= 10	132/137	0.60 (0.45-0.77)	0.68 (0.48-0.96)
Family history on diabetes			
No	1,428/1,889	1.00	1.00
Yes	622/221	3.72 (3.15-4.41)	5.24 (4.21-6.52)
Smoking status			
Never	1,279/1,121	1.00	1.00
Past	124/79	1.38 (1.03-1.85)	0.90 (0.63-1.30)
Current	355/278	1.12 (0.94-1.34)	1.04 (0.81-1.34)
Alcohol drinking			
Never	1,699/1,786	1.00	1.00
Ex-drinker	73/21	3.66 (2.24-5.96)	2.45 (1.38-4.34)
Current	271/297	0.96 (0.80-1.15)	0.78 (0.62-0.99)
Leisure physical activity			
Low	311/289	1.00	1.00
Moderate	990/1,107	0.83 (0.69-1.00)	0.95 (0.76-1.19)
High	748/695	1.00 (0.83-1.2)	0.98 (0.77-1.24)

* All the IFG individuals were excluded from this model.

† Multiple logistic regression model includes all the variables shown in this table simultaneously.

**Table 3:** Odds ratios (ORs) and 95% confidence intervals (CIs) of type 2 diabetes by risk factors of metabolic syndrome among 4,160 participants in the Shanghai Diabetes Study*

Factors	No. of cases/controls	OR (crude 95%CI)	**OR (adjusted 95%)**||
BMI (kg/m2)†			
< 24	739/950	1.00	1.00
24-28	888/866	1.32 (1.15-1.51)	1.23 (1.04-1.45)
> 28	423/294	1.85 (1.55-2.21)	1.81 (1.45-2.25)
Waist circumference†			
< 80(woman) or < 85(man)	599/828	1.00	1.00
> 80(woman) or > 85(man)	1,451/1,282	1.57 (1.38-1.78)	1.53 (1.30-1.80)
Hypertension (mmHg)‡			
No	954/1,255	1.00	1.00
Yes (>= 140/90)	1,096/855	1.69 (1.49-1.91)	1.36 (1.17-1.59)
Lipid profile§			
Triglycerides <= 1.70 mmol/L	1,200/1,434	1.00	1.00
Triglycerides > 1.70 mmol/L	850/676	1.50 (1.32-1.71)	1.55 (1.32-1.82)
HDL-C >= 1.04 mmol/L	1,314/1,573	1.00	1.00
HDL-C < 1.04 mmol/L	736/537	1.64 (1.44-1.87)	1.52 (1.23-1.79)

* All the IFG individuals were excluded from this model.

†Criteria according to the Working Group on Obesity in China, International Life Science Association. 2001

‡Criteria according to the World Health Organization/International Society of Hypertension (WHO/ISH). 1999

§ Criteria according to Chinese Cardiovascular Disease Association. 1997

||Multiple logistic regression model adjusted for age, gender, education, family history, smoking status, alcohol drinking and leisure physical activities.

## Discussion

In this large population-based case-control study of Han Chinese adults living in Shanghai, we have characterized baseline metabolic risk patterns for type 2 diabetes. Compared to controls, diabetes cases had higher prevalence of similar risk factors identified in Caucasian populations. These include lower education levels, family history of diabetes, cigarette smoking, obesity, high systolic blood pressure, glycemia and dyslipidemia. Consistent with a previous report in Caucasians, more than 70% of type 2 diabetes cases in the SDS had an age of diagnosis between 45 and 65 years [11]. Interestingly, SDS diabetes cases were five times more likely to have a family history than controls; such a magnitude of association exceeds what have previously been reported in population-based studies of Caucasians, suggesting potential stronger genetic influences of diabetes risk in Han Chinese. We have previously examined common genetic variation in several candidate gene loci (i.e., *KCNQ1, KCNJ11, CDKAL1, and FTO *genes) and confirmed some but not all of their associations with type 2 diabetes in the SDS [12-15]. Because family history represents the joint contribution of genetic susceptibility and environmental exposures, further investigation is being planned to test several emerging hypotheses in the context of gene-gene or gene-environment interactions.

Our data support the notion that obesity and central obesity in particular is a strong risk factor for type 2 diabetes, a finding that is consistent with those reported previously in various racial/ethnic populations [16,17]. Globally, it has been estimated that approximately 58% of type 2 diabetes is attributable to overweight and obesity and 90% of type 2 diabetes in Western countries is attributed to weight gain [18]. When lower cutoff points for BMI and waist circumference were used, Asians appeared to have a higher prevalence of obesity--especially central obesity--than Caucasians. Some have proposed that the BMI cutoff of 24 kg/m2 be used to define overweight in Chinese adults and that the cutoffs for waist circumference be 85 cm for men and 80 cm for women [19]. These findings also support that preventive lifestyle interventions should be targeted at lowering both BMI and central obesity in Chinese adults.

In the SDS, approximately 54% of type 2 diabetes cases were also hypertensive, which is higher than the 44% reported by the study conducted in Shanghai, 2002 [20] Moreover, a greater proportion of hypertension was also observed among diabetic individuals with normal weight, indicating the importance of maintaining normal blood pressure in non-obese diabetic patients as well.

As expected, dyslipidemia was common among participants in the SDS. High TG and low HDL-cholesterol have been reported as the main phenotypic feature of dyslipidemia in diabetes, reflecting impaired lipid metabolism [21]. Thus, strategies targeted at lowering TG levels and increasing HDL-cholesterol, particularly in non-obese individuals, may be important in reducing the risk of complications in these Chinese adults. Among those without apparent diabetes, approximately 13% had impaired fasting glucose levels and displayed a similar metabolic risk profile as diabetic cases. Such a high prevalence of IFG portends an increased diabetes incidence in the coming years because many with IFG could progress to diabetes should the levels of modifiable risk factors remain as they age.

Since type 2 diabetes is the leading cause of end-stage renal disease, preventable amputations, blindness and cardiovascular disease, public health measures are urgently needed to control the rapidly growing health and social burdens associated with this devastating disease. As a constellation of metabolic abnormalities, metabolic syndrome is a major determinant or precursor of type 2 diabetes risk, although there is as yet no consensus regarding the definitive criteria for different racial and ethnic populations [22]. The different cutoffs used for defining obesity, blood pressure, and low HDL cholesterol levels using the CDS or NCEP ATP III criteria may explain some of the observed differences in the prevalence of metabolic syndrome. Moreover, most previous studies of diabetes in China have been hospital-based or utilized convenient sampling strategies without a well-characterized source population, subjecting findings to survival and selection biases. The prevalence of MetS among controls was 15% using the CDS definition, which was slightly higher than 12% reported previously in another study of Chinese adults in 2004-2005 using the same criteria [23]. Using the ATP III criteria, the prevalence of MetS was 19%, almost identical to that reported in the Shanghai Men's Health Study[24]. Of the IFG participants and type 2 diabetes cases, 43%, 62% and 54%, 52% had the metabolic syndrome under the CDS and the NCEP ATP III criteria, respectively. However, it remains to be determined whether the CDS criteria would provide more reliable estimate of the metabolic syndrome amongst Chinese at high risk of type 2 diabetes as compared with other criteria such as the NCEP ATP III criteria [25].

Several limitations of the current study merit consideration. First, our case-control study is retrospective by design (including prevalence cases) and recall bias is thus a concern in studying risk behaviors associated with type 2 diabetes. While we plan to follow all participants in the SDS to further assess the natural history of type 2 diabetes and its complications, we note that many risk factors already identified were similar to those from well-characterized prospective cohorts of diverse racial and ethnic groups[26]. Also, we cannot completely exclude the possibility of selection bias but such bias may be limited given our response rates and population-based design.

## Conclusions

In summary, this population-based study characterizes baseline metabolic risk factors for type 2 diabetes among Chinese adults living Shanghai and generates a biological resource that enables further investigation of numerous hypotheses related to both environmental and genetic exposures in a population that has undergone a rapid socioeconomic transformation in recent years. Our initial analysis identifies the high prevalence of IFG and clustering of several important risk factors (e.g., family history and central obesity) that will be useful for the development of effective strategies for type 2 diabetes prevention in this population.

## Competing interests

The authors declare that they have no competing interests.

## Authors' contributions

HX, ZZ, LH and SL were responsible for researching the data. HX, EF, YS, SG and SL contributed to the discussion and HX, YS and SL drafted the article. All authors have read, edited, and approved the final manuscript.

## Pre-publication history

The pre-publication history for this paper can be accessed here:

http://www.biomedcentral.com/1471-2458/10/683/prepub

## Supplementary Material

Additional File 1 **Basic demographic and clinical characteristics of 4,468 participants (including 2,050 participants with diabetes) from two different districts in the Shanghai Diabetes Study, China**. This file contains a table of the basic demographic and clinical characteristics of study participants. *Total number included all participants who completed questionnaire survey. Click here for file

Additional File 2 *Multivariable-adjusted odds ratios (ORs) and 95% confidence intervals (CIs) of type 2 diabetes associated with modifiable risk factors according to three BMI levels in the Shanghai Diabetes Study. This file contains a table of the multivariable-adjusted odds ratios and confidence intervals of type 2 diabetes associated with modifiable risk factors according to three BMI levels in the Shanghai Diabetes Study. * Multivariable-adjusted odds ratios and 95% confidence intervals were from the same multiple logistic model where all the risk factors were simultaneously included. Click here for file
