# Supplementary material for: Prevalence and clustering of metabolic risk factors for type 2 diabetes among Chinese adults in Shanghai, China
Source: BMC Public Health. 2010 Nov 9;10:683. doi: 10.1186/1471-2458-10-683 (PMC2989965; doi:10.1186/1471-2458-10-683)
Supplement: Additional File 1 — Basic demographic and clinical characteristics of 4,468 participants (including 2,050 participants with diabetes) from two different districts in the Shanghai Diabetes Study, China. This file contains a table of the basic demographic and clinical characteristics of study participants. *Total number included all participants who completed questionnaire survey. [file 1471-2458-10-683-S1.DOC]

| **Characteristics** | **Baoshan Area** | **Pudong Area** | **P value** |
| --- | --- | --- | --- |
| **Total participants** ***, n** | **2,705** | **1,763** |  |
| Age (years), n (%) |  |  |  |
| 40-49 | 220 (8.1%) | 159 (9.0%) | <0.0001 |
| 50-59 | 1,012 (37%) | 649 (37%) |  |
| 60-69 | 1,014 (38%) | 519 (29%) |  |
| ≥70 | 459 (17%) | 436 (25%) |  |
|  |  |  |  |
| Sex (female), n (%) | 1,750 (65%) | 1,128 (64%) | 0.63 |
| Education (years), n (%) |  |  |  |
| 0-6 | 592 (22%) | 531 (30%) | <0.0001 |
| 7-9 | 1,479 (55%) | 1,079 (61%) |  |
| ≥10 | 613 (23%) | 153 (8.7%) |  |
| Participants with diabetes, n | 1,137 | 913 |  |
| Age at diagnosis (years), n (%) |  |  |  |
| <45 | 115 (11%) | 107 (12%) | 0.26 |
| 45-54 | 390 (37%) | 304 (35%) |  |
| 55-65 | 393 (37%) | 313 (36%) |  |
| >65 | 153 (15%) | 151 (17%) |  |
| Duration of disease (years), n (%) |  |  |  |
| <5 | 332 (32%) | 217(25%) | 0.004 |
| 5-9 | 345 (33%) | 299 (34%) |  |
| 10-15 | 246 (23 %) | 220 (25%) |  |
| >15 | 128 (12%) | 139 (16%) |  |
| Family history on diabetes | 362 (32%) | 273 (30%) | 0.35 |
| Hypoglycemic medication use | 887 (78%) | 739 (84%) | 0.001 |
