# Supplementary material for: Prevalence and clustering of metabolic risk factors for type 2 diabetes among Chinese adults in Shanghai, China
Source: BMC Public Health. 2010 Nov 9;10:683. doi: 10.1186/1471-2458-10-683 (PMC2989965; doi:10.1186/1471-2458-10-683)
Supplement: Additional File 2 — *Multivariable-adjusted odds ratios (ORs) and 95% confidence intervals (CIs) of type 2 diabetes associated with modifiable risk factors according to three BMI levels in the Shanghai Diabetes Study. This file contains a table of the multivariable-adjusted odds ratios and confidence intervals of type 2 diabetes associated with modifiable risk factors according to three BMI levels in the Shanghai Diabetes Study. * Multivariable-adjusted odds ratios and 95% confidence intervals were from the same multiple logistic model where all the risk factors were simultaneously included. [file 1471-2458-10-683-S2.DOC]

| **Variables** | **Adjusted ORs (95% CI) stratified by BMI** | | | | | | |
| --- | --- | --- | --- | --- | --- | --- | --- |
|  | **BMI<=24** | **24 <BMI< 28** | | | **BMI >= 28** | | |
| Age(years) |  |  |  |  |  |  |  |
| 40-49 | 1.00 | 1.00 |  |  | 1.00 |  |  |
| 50-59 | 1.85 (1.21-2.83) | 0.89 (0.58-1.37) | | | 1.64 (0.81-3.30) | | |
| 60-69 | 3.04 (1.95-4.73) | 1.96 (1.27-3.02) | | | 3.11 (1.54-6.31) | | |
| >=70 | 5.05 (3.07-8.32) | 3.47 (2.15-5.59) | | | 4.70 (2.18-10.1) | | |
| Sex |  |  |  |  |  |  |  |
| Female | 1.00 | 1.00 |  |  | 1.00 |  |  |
| Male | 1.62 (1.18-2.22) | 1.21 (0.89-1.65) | | | 0.90(0.55-1.47) | | |
| Education (years) |  |  |  |  |  |  |  |
| 0-6 | 1.00 | 1.00 |  |  | 1.00 |  |  |
| 7-9 | 0.57 (0.42-0.77) | 0.83 (0.63-1.09) | | | 0.76 (0.51-1.12) | | |
| >=10 | 0.41 (0.29-0.60) | 0.66 (0.46-0.94) | | | 0.49 (0.27-0.91) | | |
| Family history on diabetes |  |  |  |  |  |  |  |
| No | 1.00 | 1.00 |  |  | 1.00 |  |  |
| Yes | 5.40 (4.18-7.15) | 5.35 (4.00-7.16) | | | 4.35 (2.71-7.00) | | |
| Smoking status |  |  |  |  |  |  |  |
| Never | 1.00 | 1.00 |  |  | 1.00 |  |  |
| Past | 1.19 (0.67-2.13) | 0.72 (0.40-1.27) | | | 2.15 (0.92-5.01) | | |
| Current | 1.07 (0.73-1.58) | 1.29 (0.90-1.86) | | | 1.79 (0.95-3.37) | | |
| Alcohol drinking |  |  |  |  |  |  |  |
| Never | 1.00 | 1.00 |  |  | 1.00 |  |  |
| Ex-drinker | 1.55 (0.70-3.44) | 5.06 (1.82-14.1) | | | 1.41 (0.41-4.87) | | |
| Current | 0.66 (0.46-0.95) | 0.68 (0.49-0.95) | | | 0.94 (0.52-1.69) | | |
| Leisure physical activity |  |  |  |  |  |  |  |
| Low | 1.00 | 1.00 |  |  | 1.00 |  |  |
| Moderate | 1.17 (0.93-1.48) | 1.10 (0.88-1.37) | | | 1.12 (0.79-1.58) | | |
| High | 0.77 (0.20-3.02) | 0.82 (0.18-3.68) | | | 1.20 (0.24-6.02) | | |
| Waist-hip ratio |  |  |  |  |  |  |  |
| <=0.75 | 1.00 | 1.00 |  |  | 1.00 |  |  |
| 0.75-0.85 | 2.04 (1.04-4.00) | 1.98 (0.77-5.08) | | | 1.10 (0.18-6.71) | | |
| >=0.85 | 3.09 (1.56-6.10) | 4.03 (1.59-10.22) | | | 2.23 (0.38-13.2) | | |
| Hypertension (mmHg) |  |  |  |  |  |  |  |
| No | 1.00 | 1.00 |  |  | 1.00 |  |  |
| Yes(>=140/90) | 1.35 (1.07-1.70) | 1.22 (0.98-1.51) | | | 1.22 (0.87-1.72) | | |
| Lipid profile |  |  |  |  |  |  |  |
| Triglycerides <=1.70mmol/L | 1.00 | 1.00 |  |  | 1.00 |  |  |
| Triglycerides >1.70mmol/L | 1.17 (0.90-1.52) | 1.21 (0.97-1.50) | | | 1.29 (0.92 -1.81) | | |
| HDL-C>=1.04mmol/L | 1.00 | 1.00 |  |  | 1.00 |  |  |
| HDL-C<1.04mmol/L | 1.33 (1.01-1.75) | 1.42 (1.12-1.79) | | | 1.01 (0.70-1.44) | | |
